# Pleomorphic Giant Cell Carcinoma of the Pancreas with Hepatic Metastases—Initially Presenting as a Benign Serous Cystadenoma: A Case Report and Review of the Literature

**DOI:** 10.1155/2010/627360

**Published:** 2010-12-20

**Authors:** Petrou Athanasios, Papalambros Alexandros, Brennan Nicholas, Karles Dimitrios, Bramis Kostantinos, Manzelli Antonio, Papalambros Efstathios

**Affiliations:** ^1^Department of Hepatobilary Surgery, Churchill Hospital, Oxford OX3 7LJ, UK; ^2^Department of Pathology, Medical School, University of Athens, Greece; ^3^First Department of Surgery, Medical School, University of Athens, Greece; ^4^Medical School, University of Athens, Greece

## Abstract

*Introduction*. Pleomorphic giant cell pancreatic cancer is a very rare and aggressive pancreatic neoplasm. A case of pleomorphic giant cell pancreatic cancer presenting as a cystic lesion and in association with a serous cystadenoma presents a unique case which has not been described before. *Case Presentation*. A 44-year-old alcoholic man presented with abdominal pain, vomiting, and weight loss. Initially, imaging suspected a pancreatic pseudocyst measuring 4.2 cm. Endoscopic ultrasound- (EUS-) guided fine-needle aspiration revealed a serous cystadenoma. With conservative intervention only (fluid resuscitation, analgesia, and antiemetics) the patient improved and was discharged under close observation. Follow-up scan at four months revealed minimal change. Three months later, he was admitted acutely. Repeat scans demonstrated mild cyst enlargement with new liver lesions. Laparoscopic biopsy revealed pleomorphic giant cell carcinoma with the organ of origin the pancreas. *Conclusion*. This unusual case highlights the challenges in managing pancreatic cystic lesions and emphasizes the importance of considering less common forms of pancreatic cystic masses when the findings are atypical for the presentation. Surgical excision in these cases over conservative steps may be the most appropriate management.

## 1. Introduction

Giant cell pancreatic cancer was first described by Sommers and Meissner in 1954 [1]. In the literature, these rare tumours have been divided into two subtypes: osteoclast-like giant cell and pleomorphic giant cell carcinoma of the pancreas. Although a number of reviews have shown possible prognostic differences between these two subtypes, the most recent World Health Organisation (WHO) classification places the neoplams in the same category, undifferentiated carcinoma with osteoclast like giant cells [2]. In any case, unless detected early, the majority of cases have a very poor prognosis often worse than pancreatic adenocarcinoma [3]. In this report, we review the literature in the area and present a unique case of a patient with known alcohol abuse who developed metastatic pleomorphic giant cell cancer of the pancreas within months of a diagnosis of a pancreatic serous cystadenoma. The case also highlights the challenges in managing pancreatic cystic lesions and emphasizes the importance in considering rare forms of pancreatic cystic masses when the findings are atypical for the presentation.

## 2. Case Report

A 44-year-old man, who was a known alcoholic, presented to the First Department of Surgery, University of Athens with symptoms of epigastric abdominal pain, vomiting, and weight loss. Clinical examination demonstrated mild tenderness throughout, but no masses were noted. Biochemical analysis revealed Alanine aminotransferase (ALT) 172 U/L, Gamma-glutamyl transpeptidase (*γ*-GT) 163 U/L, alkaline phosphates (ALP) 464 U/L, and C-reactive protein (CRP) 84.90 mg/L. The serum levels of various tumour markers were not increased: Alfa Feto Protein (AFP) 1.6, Carbohydrate Antigen 19-9 (CA19-9) 12.3, Carbohydrate Antigen 72-4 (CA72-4) 1.8, and Carcinoembryonic Antigen (CEA) 3.6.

A subsequent computed tomography (CT) scan revealed the presence of a 4 cm × 4.2 cm cystic lesion in the body and tail of the pancreas. There was also a minor increase in diameter of the peripheral segment of the pancreatic duct and disseminated damage of the pancreatic parenchyma, suggestive of multiple episodes of pancreatitis in the past. There was no ascites or any other suspicious findings from the scan. Fine-needle aspiration biopsies with endoscopic ultrasound guidance (EUS) of the pancreatic lesion were performed. The biochemical analysis was positive for amylase (23,000 U/L), and cytology was consistent with the diagnosis of a serous cystadenoma. Tumour markers (CEA, AFP, and CA 19-9) were negative. The patient's symptoms subsequently improved with conservative management only: fluid resuscitation, analgesia, and antiemetics. Drainage of the cyst itself was not performed. Six days after admission, the patient was discharged with outpatient followup with repeat scanning. Four months followed without symptoms, and a second CT scan indicated only a small increase (4 cm × 4.5 cm) in the pancreatic cystic lesion. No other abnormalities were detected from the scan.

**Figure 1:**
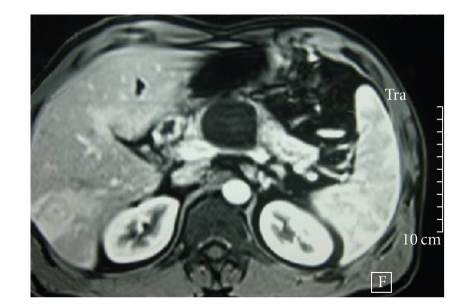
Pancreatic lesion and liver metastases on CT.

**Figure 2:**
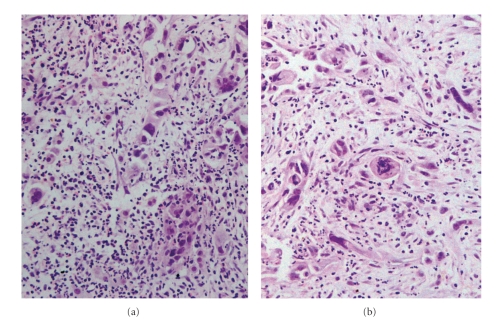
Histological section of the liver biopsy that demonstrates infiltration by a giant cell carcinoma with a diffuse growth pattern. The neoplastic cells line also pseudospaces due to lack of cohesiveness. (H & E counterstain, magnification x200).

**Figure 3:**
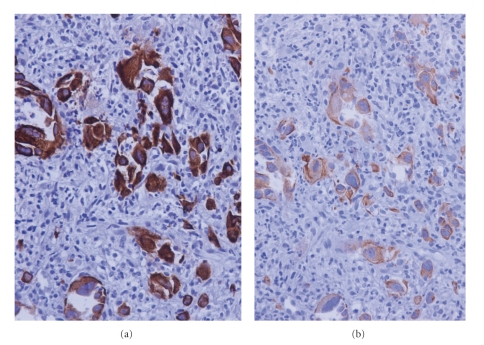
Representative immunohistochemical results. Cytokeratin 7 (a) and cytokeratin 19 (b). DAB immunohistochemistry performed by an indirect streptavidin-biotin-peroxidase method on paraffin section counterstained with haematoxylin. (magnification x400).

Three months later the patient presented acutely with recurrence of severe epigastric abdominal pain. Biochemical analysis again demonstrated deranged liver function and elevated inflammatory markers. Tumour markers were negative. A repeat CT in addition to Magnetic Resonance Imaging (MRI) and Magnetic Resonance Cholangiopancreatography (MRCP) revealed only marginal enlargement in the pancreatic cystic lesion, but now there were also multiple lesions in the liver ranging from a few milimetres to 2.5 cm in diameter (Figure 1). The patient underwent laparoscopy and biopsy which revealed infiltration of the liver tissue by a giant cell carcinoma. The neoplastic cells were anaplastic or spindle often acquiring giant cell features. The growth pattern was diffuse with pseudospaces, and the stroma was loose and abundant with inflammatory infiltrates (Figures 2(a) and 2(b)). The neoplastic cells showed cytokeratin 7 (Figure 3(a)) and cytokeratin 19 (Figure 3(b)) immunopositivity. The morphological and immunohistochemical features from the hepatic, pancreatic, and lymph node biopsies revealed the diagnosis of pleomorphic giant cell carcinoma, with the organ of origin the pancreas. At this advanced stage, surgical resection was not possible and the patient died four months later.

## 3. Discussion

Cystic lesions of the pancreas are an increasingly common finding with modern radiological investigations, although pancreatic cystic neoplasms remain rare and account for only 10%–15% of these cysts [4, 5]. Once identified, the initial step in managing cystic lesions is differentiating a pancreatic pseudocyst from a cystic neoplasm. A careful review of the clinical background of the patient is paramount, with previous documented pancreatitis or identifiable risk factors for pancreatitis (chronic alcohol consumption, history of gall stones, or a strong family history of pancreatitis) an essential starting point. If these factors are present the cystic lesion is more likely a pseudocyst, but it may also be the first presentation of a neoplastic lesion. The patient demographics, and the cyst size, site, and quantity provide valuable information in predicting the nature of the lesion [5]. Ultimately a combination of CT, MRI, MRCP, Endoscopic retrograde cholangiopancreatography (ERCP), or EUS with biopsy provides the diagnosis in most cases, with EUS the most fashionable approach at present [6–8]. The patient in this report was a 44-year-old male with known excess alcohol consumption, and CT findings are consistent with previous pancreatitis. Although the clinical features were suggestive of a pseudocyst, an EUS with biopsy was performed and while the amylase was elevated, the cytology suggested a serous cystadenoma. 

Serous cystadenomas are largely benign lesions which present more frequently in middle aged/elderly females without a history of pancreatitis are evenly distributed throughout the pancreatic gland and have a low amylase level and low tumour markers (specifically CEA) [9, 10]. The clinical features in our case did not conform to these findings. It is worthwhile noting however that the diagnostic accuracy of CT for pancreatic cysts has been reported to range from 20–90% and the sensitivity for analyzing pancreatic cystic fluid shows a range from 50–93% [11–13]. In this instance, the patient clinically improved and with cytology demonstrating a serous cystadenoma; close observation was deemed the most appropriate management. Interestingly, Tseng et al. recommend excision of large (>4 cm) serous cystadenomas irrespective of symptoms, which goes against the management of this 4.4 cm cyst [14].

Although serous cystadenomas are considered effectively benign, there have been a number of single-case reports highlighting the presence of malignant features [15, 16]. However, there are no reported cases in the English literature of a coexistent serous cystadenoma and giant cell pancreatic cancer, as was the case here. 

Giant cell pancreatic cancer is a rare neoplasm, characterised by the presence of giant cells, hypervascularity, and an inflammatory response [17]. It accounts for 2%–12.8% of all cases of pancreatic malignancies, and despite active intervention, patients usually die within months of diagnosis [17]. The neoplasm has been subdivided into two groups, osteoclast-like and pleomorphic giant cell pancreatic cancer. Indeed, a third grouping, known as mixed type, has highlighted the possibility that these tumours may indeed represent a morphological spectrum with osteoclast-like giant cell tumours at one end and pleomorphic giant cell tumours at the other [18]. Classic osteoclast-like giant-cell tumours have a predominant population of osteoclast-like giant cells and abundant hemosiderin granules whereas pleomorphic giant cell pancreatic neoplasms have more pleomorphic multinucleated giant-cells and mononuclear cells [19].

The clinical features of pleomorphic giant cell carcinoma are comparable to those of pancreatic adenocarcinoma with abdominal pain and weight loss the most prevalent [17, 20]. Cancer site has a role to play here, with head of pancreas cancers presenting more frequently with jaundice. Although there does not appear to be a preferred pancreatic site, even though some studies report higher prevalence in the body and tail. The mean age of onset is 65 years, and there appears to be a male predominance. Elevated inflammatory markers are present in the majority of cases, and CT findings often show large irregular hypodense masses (majority >6 cm) [17, 19, 20]. The survival range for pleomorphic giant cell pancreatic cancer ranges from several weeks in advanced unresectable disease to 25 months [21, 22]. The osteoclast-like giant cell variant may have a better prognosis (due to reduced prevalence of metastasis), but the evidence for this is inconclusive [20, 22, 23].

There have been at least two reported cases of osteoclast-like giant cell pancreatic tumours presenting as pseudocyst lesions and a similar number as mucinous cystadenomas [24–26]. There has been one case of a mixed (osteoclast-like and pleomorphic) giant cell pancreatic cancer presenting as a pseudocyst [27]. There have been no reported cases of an association between serous cystadenoma and any form of giant cell pancreatic cancer, which we report here. 

The diagnostic accuracy of CT, EUS with biopsy and cytology is quite high, depending on the papers cited [6–9]. However, it is more than possible for a neoplastic cyst to be missed on a single biopsy, which is plausible in the case here. However, considering the clinical improvement in the patient's condition, background history of alcohol excess +/− episodes of pancreatitis and stable disease on repeat scanning, the role of conservative management could be justified. It may also be suggested that in the eight months from initial diagnosis an aggressive pleomorphic giant cell cancer may have developed at or near the site of the presumed cystadenoma rather than a direct association between the two. The management of this case may have been different on reflection of the radiological and cytological findings considering the clinical background of the patient. Surgical resection at the initial presentation may have identified the neoplasm and altered the outcome for the patient. Overall the case emphasizes the challenge in managing pancreatic cystic lesions and suggests lowering the threshold for surgical resection in atypical cases.

## 4. Conclusion

The report discusses an unusual case of pleomorphic giant cell cancer of the pancreas which presented initially as a pancreatic cystic lesion and was diagnosed as a serous cystadenoma. The case highlights the challenges in managing pancreatic cystic lesions and emphasizes the importance of considering less common forms of pancreatic cystic masses when the findings are atypical with the presentation. Surgical excision in these cases over conservative steps may be the most appropriate management.
